# *Bifidobacterium infantis *strains with and without a combination of Oligofructose and Inulin (OFI) attenuate inflammation in DSS-induced colitis in rats

**DOI:** 10.1186/1471-230X-6-31

**Published:** 2006-10-28

**Authors:** Nadia Osman, Diya Adawi, Göran Molin, Siv Ahrne, Anna Berggren, Bengt Jeppsson

**Affiliations:** 1 Dept of Food Technology, Engineering and Nutrition, Lund University, Sweden; 2 Dept of Surgery University Hospital Malmö, Lund University, 205 02 Malmö, Sweden; 3 Probi AB, Lund, Sweden

## Abstract

**Background:**

Pathogenesis of inflammatory bowel disease is thought to be through different factors and there is a relationship between the gut flora and the risk of its development. Probiotics can manipulate the microflora in chronic inflammation and may be effective in treating inflammation. *Bifidobacterium *are saccharolytic and their growth in the gut can be promoted by non-absorbable carbohydrates and its increase in the colon appears to be of benefit.

**Methods:**

Oligofructose and inulin (OFI) alone and the two *B. infantis *DSM 15158 and DSM 15159 with and without OFI, were fed to Sprague-Dawley rats for 7 days prior to colitis induction and administrations continued for another 7 days with the DSS. Colitis severity assessed using a Disease Activity Index. Samples were collected 7 days after colitis induction, for intestinal bacterial flora, bacterial translocation, short chain fatty acids (SCFAs), myeloperoxidase (MPO), cytokines (IL-1β, TNF-α, IL-10 and TGF-β) and malondialdehyde (MDA).

**Results:**

OFI alone or the *B. infantis *strains with and without OFI improved significantly the DAI and decreased colonic MPO activity. Colonic tissue IL-1β decreased significantly in all treated groups except *B*. *infantis *DSM 15158. MDA decreased significantly in *B*. *infantis *DSM 15159 with and without OFI compared to colitis control. Succinic acid increased significantly in OFI group with and without DSM 15159 compared to all groups. Sum values of propionic, succinic acid and butyric acid increased significantly in all groups compare to the colitis control. Bacterial translocation to mesenteric lymph nodes decreased significantly in all groups compared to colitis control. Translocation to the liver decreased significantly in all groups compare to the colitis control and OFI + *B. infantis *DSM 15158 groups.

**Conclusion:**

Administrations of OFI and *Bifidobacterium *improve DSS-induced acute colitis and have an anti-inflammatory effect. Major differences in effect were observed between the two *B. infantis *strains as indicated in MDA and succinic acid concentration as well as bacterial translocation rate in synbiotic combinations.

## Background

The pathogenesis of inflammatory bowel disease is thought to involve different factors. The intestinal mucosal immune system has the formidable task of remaining unresponsive to the vast indigenous bacterial population, and yet be able to discriminate and eliminate pathogenic microorganisms to control infection. Studies in rodent models [1,2], suggest that abnormalities in this immuno-bacterial relationship may be a key to the pathogenesis of the human IBD, ulcerative colitis and Crohn's disease.

Although not one pathogenic factor has been established, relationship between the establishment of the gut flora and the risk of developing inflammatory bowel disease (IBD) has been suggested [3]. In IBD evidence has been found for a disturbed balance between pro-inflammatory and anti-inflammatory cytokines. Increased levels of the proinflammatory cytokines interleukin-1 (IL-1), IL-6, IL-8, and tumor necrosis factor α (TNF-α) were detected [4]. The cytokines are secreted by macrophages, lymphocytes, and polymorphonuclear neutrophils (PMNs) and the massively infiltrating of these cells are thought to contribute by producing large amounts of reactive oxygen metabolites (ROMs), such as superoxide anion, hydrogen peroxide, and hypochlorous acid [5].

Probiotics (micro-organism such as bifidobacteria and lactobacilli with purported physiological benefits) are being evaluated as an alternative means of manipulating the microflora in chronic inflammation. Laboratory studies have shown that probiotics (mainly lactobacilli) may be effective in treating inflammation [6-8]. Much less focus has been on bifidobacteria because of cost and maintenance of viability. In vitro, growth of bifidobacteria can be selectively stimulated by several carbohydrates indigestible by humans (prebiotics). In particular, the fructans inulin and oligofructose seem effective at stimulating growth of bifidobacteria selectively in vitro [9]. Consumption of both probiotic bacteria [10], and prebiotic components [11], reduces the severity of DSS-induced colitis.

To clarify the effects and the mechanisms behind we therefore investigate the efficacy of oral supplementation of *Bifidobacterium infantis *and oligofructose and inulin (OFI) as a primary prevention and therapy of colitis. We wanted especially to focus on effects on bacterial translocation, bacterial flora, inflammation, release of oxygen free radicals and bacterial fermentation.

## Methods

### Animals and experimental design

Sprague-Dawley rats (200 g) (Möllegård, Viby, Denmark) were used and divided into six groups (6 animals each), colitis control and five treatment groups. One group was treated with a commercially available preparation of oligofructose and inulin (OFI) (Raftilose Synergy 1, Orafti Active Food ingredients, Tienen, Belgium). This contains inulin with selected chain lengths from chicory that has been enriched by a specific fraction of oligofructose produced by partial enzymatic hydrolysis of chicory inulin. Four groups were treated with *Bifidobacterium infantis *(*B. infantis *DSM 15158 [= CURE19] or *B. infantis *DSM 15159 [= CURE21], Probi AB, Lund, Sweden) alone or together with OFI. The two *B. infantis *strains have been isolated from infant feces, and have been selected for their colonization capabilities and abilities to produce amino acids from inorganic nitrogen. The animals were kept at room temperature (22°C) with a controlled 12 hr light/dark cycle and free access to a standard rat chow (R3; Lactamin, Stockholm). The experimental solutions were administered orally by oro-gastric tube twice daily for 7 days before starting DSS and continued for 7 days after DSS induction (probiotics 3 ml; 3 × 10^6 ^CFU per animal, prebiotics 3 ml; 0.5 g per animal, synbiotics 3 ml; 3 × 10^6 ^CFU + 0.5 g per animal). Normal saline (3 ml) was administered in the colitis control group. Colitis was induced by 5% (w/v) DSS (MW = 36,000–50,000; ICN Biomedicals Inc., Aurora, OH) dissolved in drinking water for 7 days. Severity of colitis was assessed daily using a disease activity index (DAI). After the seventh day of induction of colitis, animals were anesthetized with a subcutaneous injection of a mixture (1:1:2) of Hypnorm (Division of Janssen-Cilag Ltd., Janssen Pharmaceutica, Beerse, Belgium) + Dormicum (F. Hoffmann-La Roche AG, Basel, Switzerland) + dH_2_O at a dose of 0.15 ml/100 g. Under aseptic technique a laparotomy was performed through a midline incision and samples were collected for bacterial microflora (cecum), bacterial translocation (arterial and portal blood, mesenteric lymph nodes, and liver), SCFAs (cecal contents), Myeloperoxidase (MPO), cytokines (IL-1β, TNF-α, IL-10 and TGF-β) and Malondialdehyde (MDA) (colon tissues). Bacterial microflora samples were placed immediately in sterile tubes containing, 3 ml of transport medium, whereas bacterial translocation samples were placed in sterile tubes containing, 2 ml for blood, and, 6 m1 for segments [12]. Samples for cytokines, MPO, MDA, and SCFAs were immediately frozen at -70°C. The experimental design was approved by the Animal Ethics Committee of Lund University and the experiments adhered to the guiding principles in the care and use of animals.

### Assessment of colitis

The severity of colitis was assessed daily using a Disease Activity Index (DAI) based on the scoring system of Murthy *et al*. [13,14], which scores body weight loss, stool consistency, and rectal bleeding. Occult blood in feces was evaluated by means of test slides, Hemoccult II (SmithKline Diagnostic, USA). The DAI clinical parameters used here are comprehensive functional measures that are somewhat analogous to clinical symptoms observed in human IBD and the scoring method has been validated by repeated studies [15].

### Bacterial translocation

Samples from the caudate lobe of liver, mesenteric lymph nodes, portal and arterial blood were placed in an ultrasonic bath (Millipore, Sundbyberg, Sweden) for 5 min and swirled on Chiltern (Therma-Glas, Gothenberg, Sweden) for 2 min. Viable counts were obtained from brain heart infusion (BHI) agar (Difco, Detroit, MI) that was incubated aerobically and under anaerobic condition (Gas Pack System, Gas Pack; Becton Dickenson Microbiology Systems, Cockeynsville, MD) at 37°C for 72 h (aerobic and anaerobic bacterial count, respectively). Violet-red bile glucose (VRBG) agar (Oxoid) was incubated aerobically at 37°C for 24 h (*Enterobacteriaceae *count). The number of colonies formed on each plate was counted and corrected for the weight of the original tissue and volume of blood. Tissue samples are expressed per gram of tissue while blood samples are expressed per milliliter of blood.

### Intestinal microflora

The samples from the cecum were placed in an ultrasonic bath and swirled on Chiltern as above. A conventional dilution procedure was done. Viable counts were obtained from BHI agar that was incubated aerobically and anaerobically at 37°C for 72 h (aerobic and anaerobic bacterial count, respectively), Rogosa agar (Oxoid) that was incubated anaerobically at 37°C for 72 h (lactobacilli count), from VRBG agar (Oxoid) that was incubated aerobically at 37°C for 24 h (*Enterobacteriaceae *count), and from the selective media, Modified Wilkins-Chalgren agar (MW; Oxoid), Modified Trypticase phytone-yeast extract agar (MTPY; ACSA, Spain) [16] and non-strictly selective Basic nutrient-poor media [17], which was modified according to the following composition per liter, (sodium acetate 10 g: ascorbic acid 10 g: (NH_4_)_2_SO_4 _5 g: K_2_HPO_4 _3 g: (KH)_2_PO_4 _3 g: tween 80 1 ml: yeast extract 0.5 g: glucose 20 g: agar 15 g and 5 ml of the mineral salt solution which composed per 250 ml of(MgSO_4_·7H_2_O 16 g: FeSO_4_·7H_2_O 0.5 g: MnSO_4_·H_2_O 0.35 g: NaCl 0.5 g) pH 6.18–6.24. They were incubated under anaerobic conditions at 37°C for 72 h (bifidobacteria count).

### Cytokines

Samples of colon were weighed and homogenized for 1 min in phosphate buffer. The homogenates were centrifuged at 10 000 g at 4°C for 5 min. TNF-α, IL-1β, IL-10 and TGF-β concentration in the supernatants were determined by ELISA using the commercially available Quantikine kit (R&D Systems, Minneapolis, MN 55413, USA). Optical densities were measured on an ELISA reader at a wavelength of 450 nm. Data were analyzed against the linear portion of the generated standard curve.

### Myeloperoxidase (MPO)

Colonic tissues were collected, weighed prior to storage at -70°C until time of assay for MPO activity. The colonic segments were homogenized in 1 ml potassium phosphate buffer (20 mM, pH 7.4) for 60 sec. Subsequently, the homogenate was centrifuged (14000 rpm, 10 min) and the pellet was resuspended in 1 ml potassium phosphate buffer (50 mM, pH 6.0) containing 0.5% hexadecyl-trimethyl-ammonium bromide. The sample was then freeze thawed once, sonicated (90 sec) and kept in water bath for 120 min (60°C). Next, the sample was centrifuged (14000 rpm, 10 min) and the MPO activity of the supernatant (20 μl) assessed in 96-well plates (Nunc, Invitrogen A/S, Taastrup, Denmark). The enzyme activity was determined spectrophotometrically at 450 nm. MPO (Sigma Chemical Co., St. Louis, MO, USA) was used as standard and values are expressed as MPO units g^-1 ^tissue.

### Lipid peroxidation

Malondialdehyde (MDA) were determined as index of lipid peroxidation, using MDA 586™ (R&D, Europe Ltd, Abingdon, Oxon, UK). Colonic segments were collected, rinsed in ice cold Dulbecco's PBS, weighed and then frozen immediately at -70°C for later evaluation. Lipid peroxidation was estimated by adding 1 ml Dulbecco's PBS with 5 mM butylated-hydroxyltoluene to the samples and then homogenized. After homogenization the samples were centrifuged at 4000 × g for 10 minutes (min) at 4°C. An aliquot (200 μl) of the standard and/or the supernatant was added to a reaction mixture containing 640 μl of N-methyl-2-phelindole, 10 μl probucol and 150 μl of 12 M hydrochloric acid. The samples were then incubated in a water bath for 60 min at 45°C and centrifuged at 10000 × g for 10 min at 4°C. The absorbance of the standard and supernatant was measured by Spectrophotometry at 586 nm. The results were expressed as nmol MDA/g tissue.

### Short Chain Fatty Acids (SCFAs)

SCFAs and organic acids (lactic, and succinic, acid) were measured by a modification of the capillary GC method by Richardson et al. [18] as described by Jensen et al. [19]. Sample preparation was done by diluting 10 times the cecal content samples with a working solution containing water and internal standard (IS) (100 mmol/l 2-Ethylbutyric acid). The diluted sample was homogenized and a 1 ml sub-sample was taken for extraction. This sample was extracted by adding 0.1 ml H_2_O, 500 μl hydrochloric acid (HCl) and 2000 μl ether followed by mixing for 30 seconds on a Vibrax at 1400 rpm, and centrifugation for 10 min at 3000 g. For every 8 samples, 2 standard mixtures were prepared by adding 0.1 ml H_2_O, 0.5 ml HCl, 100 μl working solution and 2000 μl ether to a 1 ml standard mixture. To the GC microvial was added 50 μl sample and/or standard from the ether-phase together with 10 μl N-tert-butyldimethylsilyl-N-trifluoroacetamid, to derivatize the samples and get volatile and thermal constant derivatives that are suitable for GC run. The vial with Crimp-Caps were closed and mixed once and placed on the 80°C heating block for 20 minutes. To complete the derivatisation, the samples were allowed to stand 48 hours before injection. The analysis was performed using a gas chromatograph provided with capillary column (DB-5, J&W Scientific, USA). The following temperature-program were used (70°C (3 min), 70–110°C (10°C/min), 110–270°C (20°C/min). Detector and injector temperature were 275 and 300°C, respectively, and the carrier gas was helium (1.9 ml/min). 2 μl sample volume was injected.

### Statistics

Values are presented as mean ± SEM or as median (10th, 25th, 75th, and 90th percentiles). For the DAI the difference between the groups was evaluated using Kruskal-Wallis one-way ANOVA on ranks followed by all-pairwise multiple comparison procedures (Student-Newman-Keuls Method). Bacterial translocations and intestinal microflora were evaluated by one-way ANOVA followed by all-pairwise multiple comparison procedures (Student-Newman-Keuls Method). For cytokines, MPO, and MDA the difference between the groups was evaluated using one-way ANOVA followed by multiple-comparison versus control-group Dunnett's method. Probability levels of <0.05 were considered significant (*p *< 0.05).

## Results

### Assessment of disease activity index DAI

**Figure 1:**
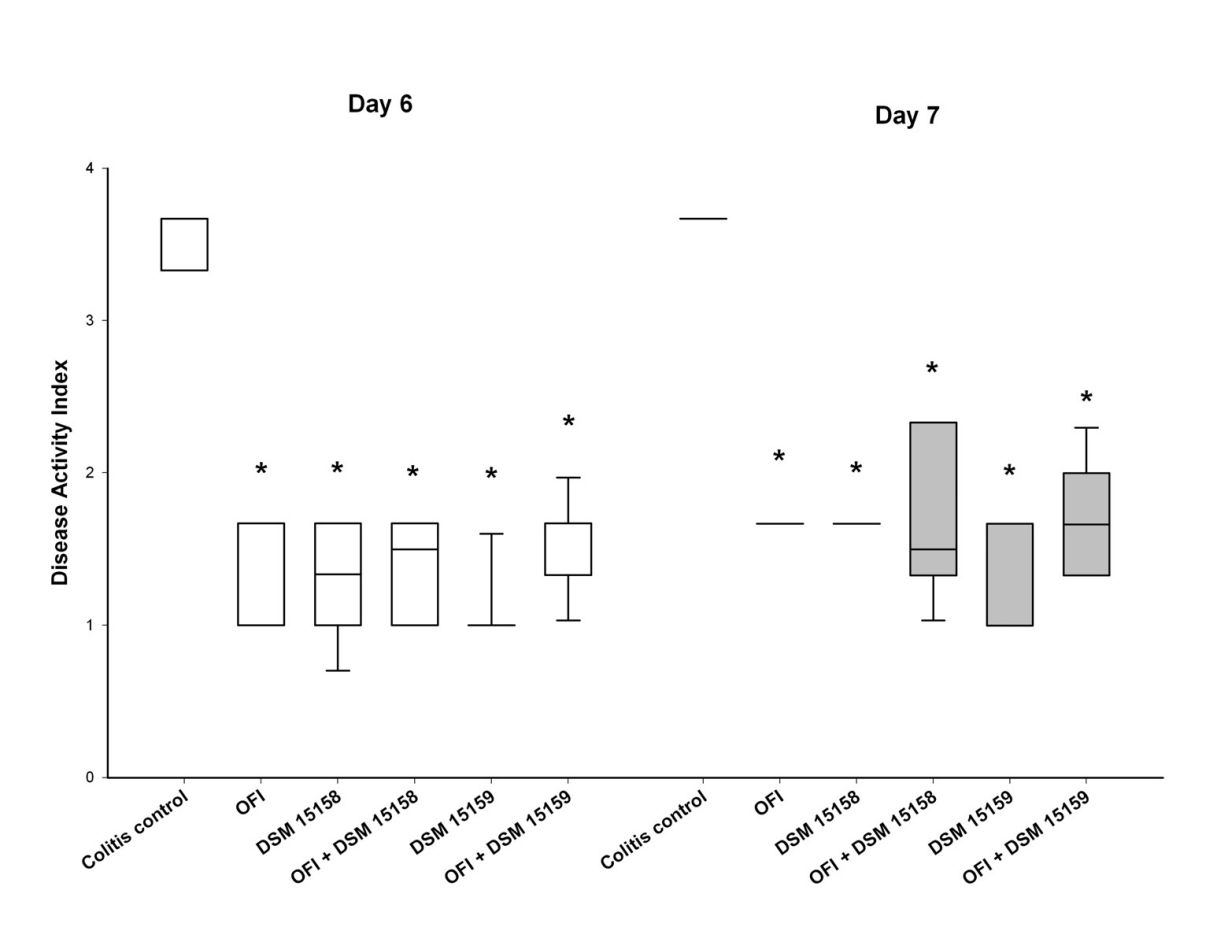
Disease activity index on day 6 and 7. Values are shown as box plots because the data were not normally distributed. The center line of each box represents the median, the top and bottom of the boxes represent the 25th and 75th percentile of the data, respectively, and the top and the bottom of the error bars represent the 10th and the 90th percentile of the data respectively. * denotes p < 0.05 compared to colitis control.

There was no mortality among the experimental groups. DAI decreased significantly on day 4 & 5 (data not shown) and day 6 & 7 in all groups compared to colitis control group (Figure 1).

### Bacterial translocation

Bacterial translocation to the liver decreased significantly in all the groups compared to colitis control and OFI + *B*. *infantis *DSM 15158 groups (Table 1). Bacterial translocation to the mesenteric lymph nods decreased significantly in all the groups compared to colitis control (Table 1).

### Intestinal bacterial flora

The bifidobacterial counts in cecum (using 2 selective media) increased significantly in the OFI groups with and without added *B. infants *compared to colitis control (8.74 ± 0.07, 8.89 ± 0.16, 8.32 ± 0.50 versus 6.82 ± 0.37 for the modified Wilkins-Chalgren agar) and (8.73 ± 0.07, 8.60 ± 0.19, 8.23 ± 0.48 versus 6.57 ± 0.37 for the modified Trypticase phytone-yeast extract agar). For the not strictly selective media it increased significantly in all groups compared to colitis control (Data not shown). There were no differences in the cecum *Enterobacteriaceae *count. The cecal count of lactobacilli increased significantly in *B*. *infantis *DSM 15159 groups with and without OFI (8.85 ± 0.24 and 9.56 ± 0.05 respectively) compared to colitis control (7.82 ± 0.47).

### Myeloperoxidase (MPO)

**Figure 2:**
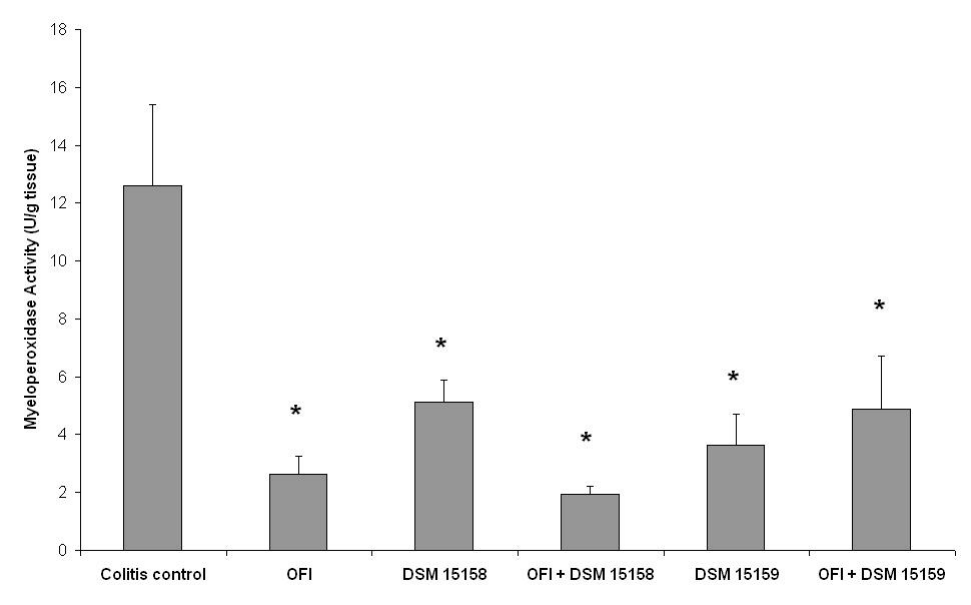
Colonic Myeloperoxidase Activities (U/g tissue). * denotes p < 0.05 compared to colitis control.

Colonic myeloperoxidase activity decreased significantly in all groups compared to the colitis control group (Figure 2).

### Cytokines

**Figure 3:**
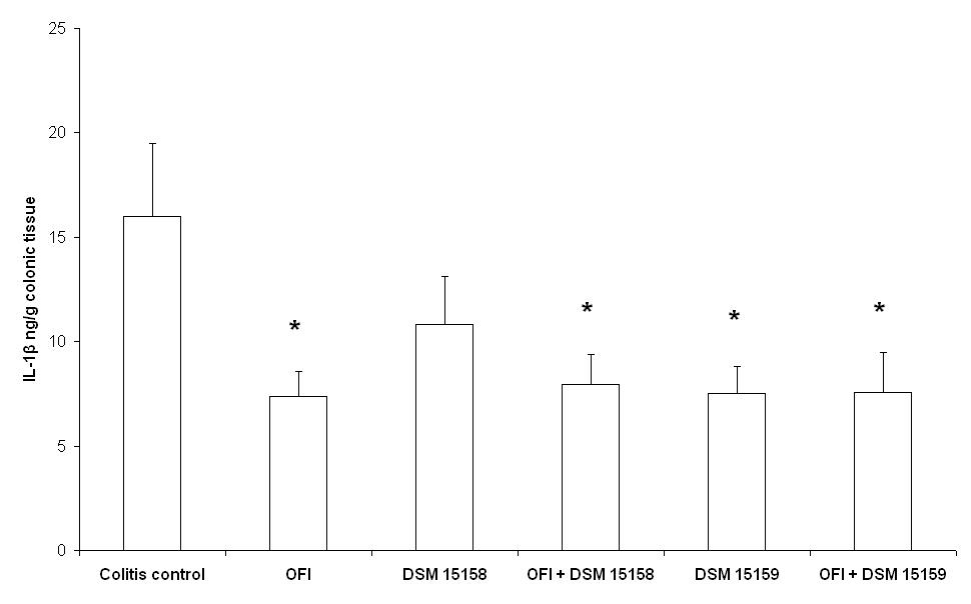
Colonic IL-1β values (pg/g tissue). * denotes p < 0.05 compared to colitis control.

Colonic tissue IL-1β decreased significantly in all treated groups except *B*. *infantis *DSM 15158 (Figure 3), while levels of TGF-β and IL-10 were maintained in all groups without significant difference (data not shown). There was no detection of TNF-α in any of the groups.

### Lipid peroxidation

**Figure 4:**
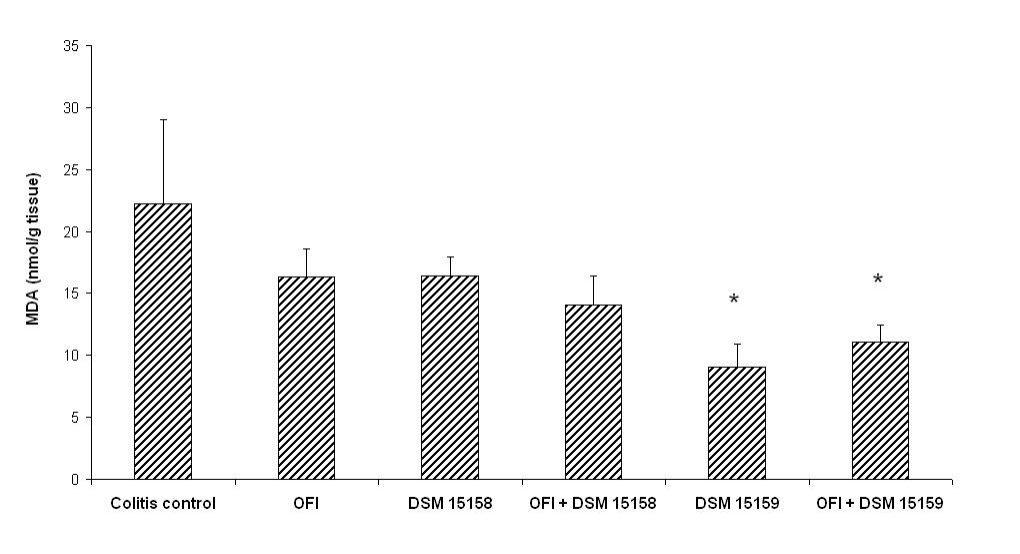
Malondialdehyde (MDA) concentration in the colon (nmol/g tissue). * denotes p < 0.05 compared to colitis control.

MDA decreased significantly in the *B*. *infantis *DSM 15159 groups with and without OFI compared to colitis control (Figure 4).

### Short-Chain Fatty Acids (SCFAs)

Succinic acid increased significantly in the OFI groups with and without DSM 15159 compared to the control and other groups (Table 2). Sum values of propionic and butyric acid increased significantly in all treated groups [5.9 (3.9–7.3), 6.1(4.1–8.6), 4.7(3.3–6.5), 5.7(4.5–6.4), 6.4(4.6–10.1)] compare to the colitis control group [3.2(2.3–3.8)]. Sum values of succinic, propionic and butyric acids also increased significantly in all treated groups [5.4 (3.6–7.0), 4.1(0.0–6.6), 3.3(1.6–5.2), 4.9(3.0–5.8), 6.0(4.0–10.0)] compare to the colitis control group [2.6(0.0–3.3)], but the OFI + *B*. *infantis *DSM 15159 was significantly higher [6.0(4.0–10.0)] than in all other groups.

## Discussion

Daily oral administration of Bifidobacteria, oligufructose and inulin produced an anti-inflammatory effect in a rat model of acute colitis induced by DSS. The effect of OFI, *B. infantis *DSM 15158 and *B. infantis *DSM 15159 both separately and in combination were characterized by a reduction in DAI. It has previously been shown in a pilot study that short term treatment of active UC with *bifidobacterium longum *and Synergy 1 resulted in improvement of chronic inflammation in patients [20]. The results of that study dose not evaluate production of SCFA or if the different bifidobacteria have similar action. We have chosen *Bifidobacterium infantis*, which was isolated from feces of healthy infants. We believe that the bacterial strains that are colonizing gut of the newborn infant have specific characteristics that may also benefit adult mucosa that is under inflammatory stress.

Both bacteria as well as the fiber component were effective in reducing colonic disease activity index, although DSM 15159 seemed superior. The same holds true for bacterial translocation where DSM 15159 was superior in reducing bacterial translocation to both liver and mesenteric lymph nodes in synbiotic combination. We found that tissue MPO activity as an index of neutrophil infiltration significantly reduced in all groups compare to the colitis control. However, the observed anti-inflammatory effect may have been reached through different mechanisms.

The increase of IL-1β in the colitis group was significantly decreased in all groups except in the *B. infantis *DSM 15158 group. The expression of IL-1 is increased in inflammatory lesions of patients with IBD (4) and IL-1 is involved in the development of DSS-induced colitis in mice [21]. The reduction of IL-1β could be achieved through different ways. Incubation of mucosal explants from Crohn's disease patients showed that certain probiotic bacteria are capable of interacting with immunocompetent cells and modulate locally the production of proinflammatory cytokines by inflamed tissue [22]. A human epithelial cell line HT29/19A, which produces the chemokines IL-8, upon stimulation with IL-1, TNF and interferon when incubated with bacterial cell suspensions showed that Gram-positive probiotic bacteria did not induce IL-8 production, whereas the non pathogenic Gram-negative strain did in a dose-dependent way [23]. It has been shown that butyrate significantly inhibits Th1-type responses and that this might explain the therapeutic effect of butyrate in IBD patients. Acetate and propionate have less marked modulatory actions, and in some cases have effects that oppose those of butyrate. A combination of the three SCFA causes a shift in the T helper lymphocyte phenotype towards a more anti-inflammatory phenotype [24], and in this study we found that succinic acid increased significantly in OFI group with and without DSM 15159 compared to the colitis control and other groups and the sum values of propionic, butyric and succinic acid increased significantly in all treated groups compared to the colitis control group. However, this might explain that the protective effects of prebiotics and probiotics could be through the production of SCFAs.

The production of TNF-α was not detected in all our groups. We can not for sure conclude or suggest that TNF-α has no role in intestinal inflammation. The lack of detection in our experiment could be due to the sensitivity of the ELISA kit. Also reports of this cytokine in inflammatory bowel disease IBD are somewhat contradictory, whereas some groups were able to demonstrate increased levels of TNF others were unable to detect it in patients with IBD (4). In another animal study, failure to detect local or systemic TNF and failure to prevent colonic inflammation with anti-TNF antiserum have pointed that TNF could not be an inflammatory mediator in DSS-induced murine colitis (25). IL-10 and TGF-β were maintained in all groups and this suggests that mediators other than them are involved in the anti-inflammatory effects. These results are in agreement with a previous study [7]. On the other hand, studies of IL-10 levels in inflamed mucosa are not conclusive. Preliminary data from previous researchers indicate that a feed back loop may exist in IBD, in which an increased production of the cytokine interferon-γ down regulates IL-10 production, therefore its level in the mucosa may be (although increased) not adequately high [26]. Topical IL-10 enema treatment of patients with ulcerative colitis unveiled that IL-10 is effective in down-regulating pro-inflammatory cytokine synthesis from IBD monocytes and lymphocytes both *in vitro *and *in vivo *[27]. In that study, equal concentrations of IL-10 appear detectable in both normal and IBD intestinal lamina propria biopsy homogenates. This seems surprising in view of other results [28,29] also argue against a general deficiency in IL-10 production in IBD. They in fact observed an increase in IL-10 producing cells and rather suggest that in IBD the production of IL-10 is dislocated and insufficient to down-regulate pro-inflammatory cytokines in the lamina propria compartment. It has also bean shown that IL-10 gene therapy is therapeutic for dextran sodium sulfate-induced murine colitis [30].

Oxidative stress has been implicated in the pathogenesis of inflammatory bowel disease. Enhanced release of reactive oxygen species (ROS) plays an important role in the pathogenesis of clinical inflammatory bowel diseases. ROS are also involved in the pathogenesis of DSS-induced colitis [31]. In the IBD patients serum lipid peroxidation products were significantly elevated when compared to control subjects, suggesting the presence of increased oxidative stress consistent with inflammatory activity [32]. It has been shown that measurements of lipid peroxidation confirm previous evidence, indicating that development of colitis is associated with a significant burst in ROS [33]. We measured MDA in colonic tissues as an assessment of oxidative stress. We found significantly decreased levels of mucosal lipid peroxidation products in the *B*. *infantis *DSM 15159 groups with and without OFI compared to the colitis group. This is consistent with findings in other studies showing an increased in lipid peroxidation products with inflammation, both in Crohn's disease and ulcerative colitis [34]. We also found that there was increase in succinic acid in *B. infantis *DSM 15159 + OFI group. Because of the pH-depended inhibition of the respiratory burst by succinate [35], this increase could cause a significant reduction in pH, thereby succinic acid may be by reducing pH further reduce neutrophils infiltration and the neutrophil respiratory burst [35].

*B. infantis *DSM 15159 with and without OFI increased significantly cecum lactobacilli count, which might suggest that the reduction of lipid peroxidation might also be caused by the formation of antioxidant factors by lactobacilli. Cecum bifidobacteria count increased significantly in OFI with and without *B. infantis *strains and this is probably due to the fact that bifidobacteria utilized OFI rapidly and increased in numbers. Wang *et al *(9) showed that oligofructose and inulin selectively stimulated growth of bifidobacteria. Bacterial translocation has been reduced by most of the groups to MLNs and liver. In MLNs the increased translocation in DSS-colitis decreased significantly in all treated groups. As for the liver, the translocation decreased significantly in all groups compare to the colitis group and OFI + *B. infantis *DSM 15158 groups. This reduction in bacterial translocation could be due to the reduction in the inflammation by the different mechanisms mentioned before. It seems that the translocation to the extraintestinal sites is affected by the administration of pre- and probiotic and this could be due to the effects on gastrointestinal tract, barrier functions and intestinal inflammation. These have been reflected by the reduction of IL-1β, MPO and MDA in the intestinal tissue, and reduction of disease activity index.

## Conclusion

Major differences in effect were observed between the two tested strains of *B. infantis *with regards to MDA, succinic acid concentration and translocation rate. The mechanisms by which probiotics, prebiotics and synbiotics work and affect the colitis induced by DSS could be through direct and indirect effects on microflora ecology, barrier functions and cellular proliferation, and production of different substances e.g. SCFAs, which could affect the local and systemic immunity and lipid peroxidation.

## Competing interests

The authors declare that they have no competing interests. Dr Berggren is affiliated with Probi AB.

## Authors' contributions

NO contributed in designing the study with other authors, performed experimental studies and analysis, data analysis, prepared and wrote the manuscript. DA study design contribution, performance of experimental studies, input to manuscript preparation. GM study design, co-ordination and supervision of the study, input to manuscript preparation. SA study design, co-ordination and supervision of the study, input to manuscript preparation. AB performance of SCFAs analysis. BJ study design, co-ordination and supervision of the study, input to manuscript preparation.

## Pre-publication history

The pre-publication history for this paper can be accessed here:



## Figures and Tables

**Table 1:** Bacterial translocation count to the liver and mysenteric lymph nodes (MLNs) (log CFU/g tissue) and Incidence of translocation

	Aerobic	Anaerobic	*Enterobacteriaceae*
**Liver**			
Colitis control	2.72 ± 0.77 (4/6)	3.58 ± 0.33 (5/6)	1.89 ± 0.66 (4/6)
OFI	0.38 ± 0.38 * # (1/6)	0.50 ± 0.50 * # (1/6)	0.00 ± 0.00 * # (0/6)
DSM 15158	0.00 ± 0.00 * # (0/6)	0.00 ± 0.00 * # (0/6)	0.00 ± 0.00 * # (0/6)
OFI + DSM 15158	3.14 ± 1.01 (4/6)	3.29 ± 1.06 (4/6)	2.98 ± 0.73 (4/6)
DSM 15159	0.55 ± 0.55 * # (1/6)	0.57 ± 0.57 * # (1/6)	0.00 ± 0.00 * # (0/6)
OFI + DSM 15159	0.00 ± 0.00 * # (0/6)	0.00 ± 0.00 * # (0/6)	0.00 ± 0.00 * # (0/6)

**MLNs**			
Colitis control	2.61 ± 0.76 (4/6)	2.71 ± 0.80 (4/6)	1.73 ± 0.73 (3/6)
OFI	0.00 ± 0.00 * (0/6)	0.00 ± 0.00 * (0/6)	0.00 ± 0.00 * (0/6)
DSM 15158	0.00 ± 0.00 * (0/6)	0.00 ± 0.00 * (0/6)	0.00 ± 0.00 * (0/6)
OFI + DSM 15158	0.48 ± 0.48 * (1/6)	0.00 ± 0.00 * (0/6)	0.00 ± 0.00 * (0/6)
DSM 15159	0.00 ± 0.00 * (0/6)	0.00 ± 0.00 * (0/6)	0.00 ± 0.00 * (0/6)
OFI + DSM 15159	0.00 ± 0.00 * (0/6)	0.00 ± 0.00 * (0/6)	0.00 ± 0.00 * (0/6)

* denotes p < 0.05 compared to colitis control, # denotes p < 0.05 compared to OFI + DSM 15158 group, OFI = Oligofructose and Inulin. Incidence of translocation is presented between parentheses as positive animals/total number of animals.

**Table 2:** SCFAs values (μmol/g cecal content) of all the experimental groups

SCFAs	Colitis Control	OFI	DSM 15158	OFI + DSM 15158	DSM 15159	OFI + DSM 15159
Succinic	0.0 (0.0–1.1)	4.5 (2.9–5.7) *	0.0 (0.0–0.0) # §	1.5 (1.2–1.6) # §	0.4 (0.0–4.7) # §	5.9 (4.0–6.8) *
Propionic	3.3 (3.1–3.3)	6.2 (3.6–7.0)	4.7 (3.2–5.7)	4.9 (4.7–8.8)	5.7 (5.1–5.9)	7.5 (5.9–10.3)
Butyric	2.3 (0.0–8.4)	5.5 (4.2–7.6)	6.8 (6.4–13.7)	3.3 (3.1–6.2)	5.7 (3.9–6.8)	5.9 (3.3–10.0)
Acetic	23.1 (22.7–28.1)	35.8 (26.1–47.1)	30.9 (24.7–46.3)	20.0 (18.6–25.3)	22.9 (20.7–28.3)	28.4 (20.8–36.8)
Lactic	0.0 (0.0–0.0)	1.6 (0.0–3.2)	2.5 (2.4–3.0)	0.4 (0.0–1.1)	0.4 (0.0–1.0)	2.0 (0.0–2.8)

* denotes p < 0.05 compared to colitis control; # denotes p < 0.05 compared to OFI + DSM 15159; § denotes p < 0.05 compared to OFI. Data presented as Median (25^th^–75^th ^percentile)
